# Bioinformatic *cis*-element analyses performed in *Arabidopsis* and rice disclose bZIP- and MYB-related binding sites as potential AuxRE-coupling elements in auxin-mediated transcription

**DOI:** 10.1186/1471-2229-12-125

**Published:** 2012-08-01

**Authors:** Kenneth W Berendzen, Christoph Weiste, Dierk Wanke, Joachim Kilian, Klaus Harter, Wolfgang Dröge-Laser

**Affiliations:** 1 Zentrum für Molekularbiologie der Pflanzen, Pflanzenphysiologie, Universität Tübingen, Auf der Morgenstelle 1, 72076, Tübingen, Germany; 2 Julius-von-Sachs-Institut, Pharmazeutische Biologie, Universität Würzburg, Julius-von-Sachs-Platz 2, 97082, Würzburg, Germany

**Keywords:** *Cis*-elements, *cis*-element modules, Auxin-regulated transcription, AuxRE, bZIP, MYB, MYC

## Abstract

**Background:**

In higher plants, a diverse array of developmental and growth-related processes is regulated by the plant hormone auxin. Recent publications have proposed that besides the well-characterized Auxin Response Factors (ARFs) that bind Auxin Response Elements (AuxREs), also members of the bZIP- and MYB-transcription factor (TF) families participate in transcriptional control of auxin-regulated genes via bZIP Response Elements (ZREs) or Myb Response Elements (MREs), respectively.

**Results:**

Applying a novel bioinformatic algorithm, we demonstrate on a genome-wide scale that singular motifs or composite modules of AuxREs, ZREs, MREs but also of MYC2 related elements are significantly enriched in promoters of auxin-inducible genes. Despite considerable, species-specific differences in the genome structure in terms of the GC content, this enrichment is generally conserved in dicot (*Arabidopsis thaliana*) and monocot (*Oryza sativa*) model plants. Moreover, an enrichment of defined composite modules has been observed in selected auxin-related gene families. Consistently, a bipartite module, which encompasses a bZIP-associated G-box Related Element (GRE) and an AuxRE motif, has been found to be highly enriched. Making use of transient reporter studies in protoplasts, these findings were experimentally confirmed, demonstrating that GREs functionally interact with AuxREs in regulating auxin-mediated transcription.

**Conclusions:**

Using genome-wide bioinformatic analyses, evolutionary conserved motifs have been defined which potentially function as AuxRE-dependent coupling elements to establish auxin-specific expression patterns. Based on these findings, experimental approaches can be designed to broaden our understanding of combinatorial, auxin-controlled gene regulation.

## Background

Auxin is a major plant hormone that regulates concerted plant growth as it is involved in diverse plant developmental processes [1] such as apical dominance [2], root formation [3] and growth-related tropisms [4]. In general, the manifestation of auxin-mediated responses is ascribed to the encoded activity of auxin responsive genes [5].

The transcriptional response to auxin is primarily mediated through *cis*-regulatory Auxin Response Elements (AuxREs) [6]. These elements are bound by Auxin Response Factors (ARFs) [7] that act with Aux/IAA proteins to regulate auxin dependent gene transcription, whereby Aux/IAA proteins repress ARF activity at low cellular auxin concentrations [8-10]. Auxin mediates the interaction of AUX/IAA repressor proteins with the SCF^TIR1^ auxin receptor complex that marks the repressor proteins for degradation by the 26 S proteasome. Thus, increasing auxin concentrations lead to a de-repression of ARF-TF target genes [11,12]. AuxREs were first discovered and characterized by foot-printing analysis within the *PsIAA4/5* promoter of *Pisum* sativum [13]. Later, they were shown to function in a soybean *GH3* promoter [14,15]. Although it could be demonstrated that AuxREs are sufficient to provide auxin-responsiveness [7,15,16], they have been found to occur and function as composite elements in a genuine promoter context [9,17]. Therefore, it has been postulated that AuxRE coupling elements might have a modulating effect in the transcriptional response to auxin [15].

Recently it has been pointed out, that a member of the *Arabidopsis* R2R3 MYB TF family, namely AtMYB77, interacts with AtARF7 and synergistically promotes the expression of an auxin-responsive reporter construct [18]. In line with these findings, *Arabidopsis myb77* mutant plants exhibit a down-regulation of several auxin-responsive genes which harbour multiple, putative Myb Related Elements (MREs) in their promoters [18]. In comparison to wild type (wt) plants, *myb77* and the auxin receptor mutant *tir1-1*, show a similar decrease in auxin-controlled lateral root density under potassium-deprived conditions [18]. Hence, it has been concluded that AtMYB77 plays a role in altering auxin responses during transition from nutrient-sufficient to nutrient-deficient conditions by presumably modulating the plant’s sensitivity to auxin [18].

Besides MREs, bZIP Response Elements (ZREs) have also been reported to be potential quantitative elements in auxin-mediated transcription. The well-characterised auxin-responsive soybean *GmGH3* promoter, for instance, contains three composite units, encompassing AuxREs and adjacent or partially overlapping G-box Related Elements (GREs). EMSA studies confirmed that a recombinant G-box specific basic leucine zipper (bZIP) TF can bind to these GREs [15,19]. A similar promoter organisation was found for the auxin-responsive *GmAux28* gene in which the GREs were bound by two soybean G-box binding factors, SGBF-1 and SGBF-2 [20,21], which however have not been functionally characterized, yet. In tobacco, AuxRE and GRE composed modules were located in the promoter of the *NtGH3* gene and at least two GREs were recognised by the tobacco bZIP factor NtBZI-1, that regulates *NtGH3* transcription and auxin-related growth responses [22].

Despite these observations it is yet unknown whether modules of AuxRE, ZRE and MRE *cis*-elements frequently occur in auxin-responsive promoters and, thus, may contribute to a common regulatory mechanism in auxin-mediated transcription.

To address this question, we conducted with the novel Motif Mapper *cis*-element analysis tool a genome-wide bioinformatic analysis of auxin-responsive promoters in a dicot (*Arabidopsis thaliana*) and monocot (*Oryza sativa*) model plant. These analyses confirmed that specific singular and composite motif-modules, consisting of AuxREs, ZREs, MREs or a G-box related MYC2 element, are significantly enriched in the promoters of auxin-inducible genes and in some auxin-regulated gene families. In particular, an enrichment of a GRE-AuxRE bipartite module was found. Transient protoplast transfection assays experimentally underlined the relevance of GREs as quantitative modulators of auxin-induced, AuxRE-mediated transcription.

## Results

### Promoters of *GmGH3* homologs from several monocot and dicot plant species exhibit ZRE and MRE motifs in close proximity to AuxREs

Previous studies have suggested that ZREs and MREs might play a considerable role in the regulation of some auxin-inducible *GH3* promoters [14,18,22,23]. Especially, GRE and TGA motifs (see Table 1), which are bound by G-box binding factors (GBFs) and TGA-TFs [24,25], were frequently found to reside near AuxREs. Similar observations were made for MREs with respect to the *Arabidopsis GH3.2* and *GH3.3* genes [18].

**Table 1:** **Overview of TF-binding sites used in the promoter** ***cis*-element analyses**

**Binding sites**	**Abbr.**	**Sequence**	**Putative trans-acting factors**	**References**
**bZIP related binding sites (ZREs)**				
G-box related element	GRE	BACGTV	bZIPs	[24,26]
TGA element	TGA	TGACG	bZIPs (group D)	[25]
ACTCAT element	AC	ACTCAT	bZIPs (some group S)	[27]
**B3-type related binding sites**				
AuxRE	AUX1	TGTCTC	B3-type (ARFs)	[7]
AuxRE-related element	AUX2	TGTCYS	B3-type (ARFs)	[28,29]
Sph/RY element	RY	CATGCATG	B3-type (e. g. ABI3)	[30]
**Myb/Myc related binding sites**				
Myb-related element 1	MRE1	AMCWAMC	MYBs	[31]
Myb-related element 2	MRE2	GGWTW	MYBs	[32,33]
Myc-related element	MYC2	CACATG	MYCs	[34]

*Cis*-elements with envisaged role in auxin-mediated transcription were compiled and organized into three classes: bZIP response elements (ZREs), B3-type TF-related elements (AUXs/RY) and MYB/MYC related elements (MREs/MYC2). The corresponding references are indicated.

In order to elucidate whether this phenomenon is specific for these genes or might be a general feature of early, auxin-responsive *GH3* promoters, we identified homologs of the soybean *GmGH3* in several monocot (*Oryza sativa, Sorghum bicolour, Zea mays*) and dicot (*Arabidopsis thaliana, Glycine max, Lotus japonicus, Medicago truncatula, Vitis vinifera*) plant species. For the homology search, considerably low BLAST scores (≤ 1x10^-160^) were chosen to restrict the dataset to likely ortholog and paralog candidates. On the basis of the GH3 protein sequences, a neighbour-joining phylogram was created using a *Physcomitrella patens* GH3 (PpGH3-1) as the outgroup. The corresponding *GH3* promoters (−1000 to −1 bp) were scanned using both Watson and Crick words for the consensus AuxRE motif (TGTCTC core sequence) [35], which we call AUX1, and its less stringent variant, named AUX2 (TGTCYS) [28,29]. Furthermore, the promoters were scanned for three different ZREs (GRE-, TGA- and AC-motif) found to be bound by bZIPs [24,25,27], and two MREs; MRE1 (AMCWAMC) and MRE2 (GGWTW) [31-33] (Table 1).

**Figure 1:**
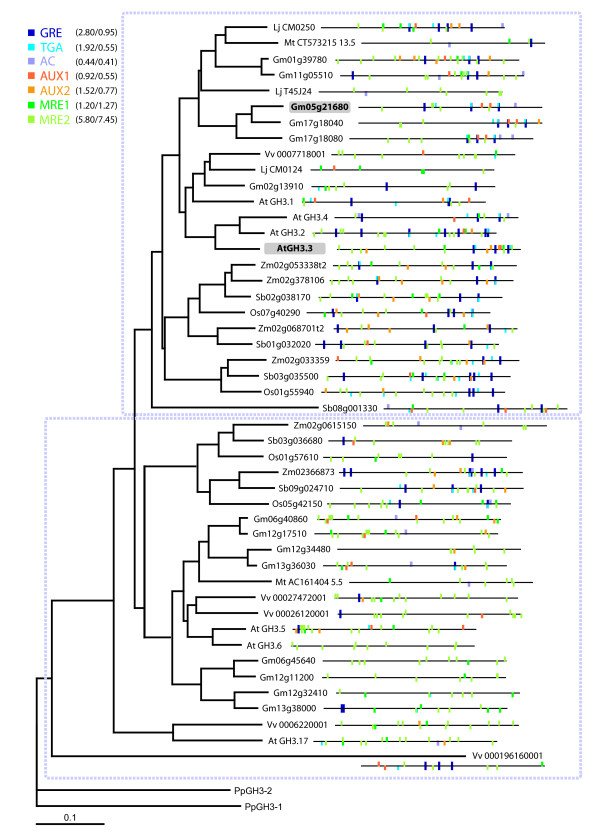
**Phylogram of GH3 homologs from various species and their associated promoter *cis*-element organisations.** The nearest neighbours of the well-characterized soybean GH3 (Gm05g21680) [36] from several plant species were compiled and the corresponding predicted protein sequences were rooted to *Physcomitrella patens* PpGH3-1 (at the bottom) to create a neighbour-joining phylogram. The −1000 bp promoter sequences of the corresponding *GH3* genes were manually co-plotted, in 5’ to 3’ orientation onto the phylogram. The location of specific ZRE, MRE and AuxRE *cis*-elements (see Table 1) are given using the presented colour code. The indicated boxes divide the phylogram into the ZRE-rich (upper section) and ZRE-poor (lower section) clade. The average number of *cis*-elements per promoter within the clades (ZRE rich/ZRE poor) is given next to the motif colour code. The position of the soybean GH3 (GmGH3) and its related *Arabidopsis* homolog (AtGH3.3), used in this study is highlighted in grey. The indicated scale reflects the number of amino acid substitutions per site.

The resulting phylogram revealed that the analysed MRE motifs were frequently distributed throughout the *GH3* promoters tested (Figure 1). Especially, the MRE2 motif occurred at a high frequency and at least once in every promoter, whereas MRE1 showed a lower overall abundance, but was still present in a relevant proportion (~ 75%).

Concerning the distribution of the ZREs, it was observed that the majority of the promoters closely related to *GmGH3* contained one or more of these elements. Only a small number of them (~ 13%) did not contain any ZRE. With respect to ZRE motif abundance, a relationship between protein homology and promoter organisation was observed. More precisely, it was possible to demonstrate that closely related GH3 proteins can be separated in two more or less distinct clades according to the occurrence of ZREs in their corresponding promoter sequences. The only exceptions were a GH3 homolog from *Lotus japonicus* (LjCM0124) and three closely related GH3s (Zm02366873, Sb09g024710, Os05g42159) which form a minor sub-clade. They were found on protein homology level within the envisaged ZRE rich or poor clades. However, regarding motif abundance they should be located in the respective other one. Moreover, it was notable that promoters from the *GH3* homologs of all tested plant species were either rich or poor of ZREs, indicating that these two groups of *GH3s* might be differently regulated. A closer inspection of ZRE abundance showed that GRE- and TGA-motifs were the most prominent ZREs, whereas the AC-motif occurred relatively rare in the *GH3* promoters, albeit with a higher frequency in the ZRE rich clade. Finally, it should be pointed out that ZREs and MREs were often found close to AuxRE motifs within a flexible spacing of around 100 bps.

In summary the results from this promoter *cis*-element analysis demonstrate that defined *GH3* subclasses exhibit a conserved distribution and abundance of specific AuxRE, ZRE and MRE motifs in their promoters that is congruent with their protein phylogeny. This indicates that GRE, TGA and MRE *cis*-elements might co-operate with AuxREs in the regulation of specific auxin-responsive *GH3* genes. In order to examine whether this clustering of AuxRE, ZRE and MRE motifs is conserved in auxin-responsive promoters, we analysed their abundance and distribution on a genome-wide scale.

### A real randomization algorithm was designed to detect significantly enriched or depleted *cis*-elements and combined motif-modules in selected promoter datasets

To identify enriched motifs and motif combinations in specific promoter datasets, appropriate control promoter datasets are required. Therefore, we designed a randomization algorithm for the Motif Mapper software [37] which enabled statistical evaluation of motif enrichment in a specific promoter dataset compared to a randomly composed dataset. The determination of significant enrichment or depletion of certain *cis*-elements or *cis*-element combinations in a given dataset is quite challenging. The difficulty arose in how to define the number of *cis*-elements and modules as accurate as possible. Many algorithms are available capable of searching for *de novo* or user defined modules [38]. Some of them function reliably with metazoans, such as TOUCAN2 [39], while other algorithms require clusters of co-expressed genes. However, we intended to develop a simple, but effective method for testing the significant occurrence of various motifs and modules at the same time in unprocessed lists of genes without prior clustering. Therefore, we decided to test for motif and module enrichment in comparison to a real randomized, full genomic promoter dataset for each individual species without superimposed modelling. The features of this algorithm were integrated into the graphical interface version of Motif Mapper (see Methods) and are presented in the following section.

#### Module description

**Figure 2:**
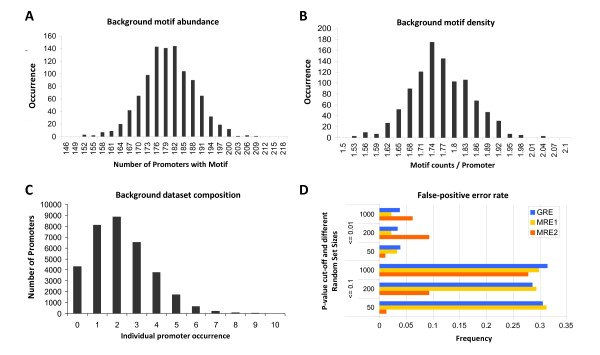
**Output of the applied randomization algorithm.A**) Background motif abundance of the GRE motif. The parameter “number of promoters with a motif” was exemplarily determined for the GRE motif in several randomized promoter datasets (1000 random sets of 304 genes) and its distribution is visualised in the given histogram. Experimental datasets which exhibit a significant enrichment or depletion (for e.g. the GRE motif) should display a respective significant shift in their position in the background distribution. **B**) Background motif density of the GRE motif. The parameter “motif counts per promoter” was exemplarily determined for the GRE motif in several randomized promoter datasets (1000 random sets of 304 genes) and the average number of motif counts per promoter is visualised in the given histogram. Experimental datasets which exhibit a significant enrichment or depletion should display a respective significant shift in their position in the background distribution. **C**) Background dataset composition. Illustration of the number of times a promoter was randomly drawn to participate in the reference dataset. The algorithm draws individual promoters from genomic datasets only once or twice (average from 1000 dataset randomizations), indicating that only very limited redundancy is present in the background dataset modelling. **D**) False-positive error rate estimations for parameter I. The error rates for genomic frequent and infrequent motifs used in this study are given. It was calculated for each motif in differently sized random datasets (50, 200 and 1000). The approach performed well on all dataset sizes, however becomes inaccurate if the background distribution of the motif (e.g. MRE2) is not approximately Gaussian. The given false-positive error frequency is the number of p-value calls (x-axis) under a set α value (y-axis) observed in 1000 random calculation repetitions.

We used word matching while allowing alternative bases to be represented by International Union of Pure and Applied Chemistry (IUPAC) letters [40]. Modules can be composed of any number of motifs, with any defined or flexible spacing between them. Previous work has suggested that some *cis*-motifs have a 5’ to 3’ bias with respect to the transcriptional start site of a gene [41,42] but the full significance for TF recognition is still unknown. In order to explore if composite modules could also have a 5’ to 3’ bias, *cis*-element modules were analysed in both orientations, while allowing the single embedded *cis*-motifs to be identified on both strands as Watson or Crick words. In contrast to other bioinformatic approaches, there is no need to extrapolate the relationships between multiple motifs a priori. For any set of genes of size n, the algorithm extracts a random set of size n for any number of repetitions. We found that 1000 random extractions yield reliable results in a reasonable amount of time. Using this approach, it is possible to calculate the significance of four parameters simultaneously: (I) the number of promoters with a motif (Figure 2A); (II) the average number of motifs per promoter (Figure 2B); (III) the total number of motifs and (IV) the variance of the average number of motifs per promoter, which indicates whether or not a specific motif is equally abundant within the promoters of a given promoter dataset. The algorithm output includes three dataset values: (a) the actual input dataset, (b) the average of the random datasets and (c) the obtained p-values. The random sampling is sufficient to yield Gaussian background distributions from which p-values can be effectively calculated using a Z-score. Since a complete genomic population distribution is present, this method can be used to calculate for both motif enrichment and depletion of normally and near-normally distributed *cis*-elements. Some dataset parameters for the GRE motif are shown for illustration in Figure 2 A, B with respect to its random background distribution.

#### False-positive error rate

To evaluate the quality of the randomization process, the algorithm enables the user to return both the randomization indices (Figure 2A, B, C) and the values of the four parameters used for calculating the p-values. The randomization algorithm was able to deliver Gaussian distributions from the background (Figure 2A, B) and overall, most individual promoters were called only 1 to 3 times (Figure 2C) indicating that the sampling of the genomic background dataset was sufficient. The false-positive error rate was determined by screening 1000 times, randomly composed datasets of various sample sizes (50, 200 and 1000 promoters) for various motifs and calculating the probability that a given motif is termed enriched by mistake. Figure 2D shows the false-positive error rate for two p-value cut-offs (0.1 and 0.01) for the GRE and two MRE motifs in the *Arabidopsis* genomic promoter dataset. The individual sequence of a motif and its presence in the background population can strongly affect the false-positive error rate. For example, the MRE2 motif exhibited a high false-positive error rate for large dataset sizes when scoring parameter I (“promoters with a motif”). This is because we employ a Gaussian Z-statistic presumption which requires that the underlying background distribution for the analysed motifs is Gaussian, which is for the highly abundant MRE2 in the *Arabidopsis* and rice genome not the case. For that reason we included further parameters as e.g. parameter II (“motif density”) which exhibits for MRE2 (and likely also for the vast majority of abundant motifs) a Gaussian distribution pattern. Nevertheless, we recommend to adhere for uncorrected p-values to an α ≤ 0.01 or less for this parameter.

#### The number of promoters with a motif is in general a reliable significance parameter

It is in principle assumed that a set of genes which are co-operatively regulated by the same type of TF require that their associated promoters contain the corresponding TF-binding site at least once. Furthermore, we can assume that this *cis*-element should be in either all or a significant proportion of the TF regulated promoters. In accordance with this, we observed that parameter I (“number of promoters with a motif”) carried the most significant relation for *cis*-element enrichment or depletion in a specific dataset. In the exceptional case that a motif was highly abundant in the analysed genomic promoter dataset, e. g. MRE2 as described in the previous section, parameter II (“number of motifs per promoter”) was consulted to meet the Gaussian presumption and to obtain meaningful results regarding an enrichment or depletion of such a *cis*-element. For that reason, we drew our attention in the majority of *cis*-element distribution analyses to parameter I as the most relevant observation parameter but also presented parameter II as positive or negative motif density for abundant motifs.

Bioinformatic *cis*-element analysis of auxin-responsive promoters from *Arabidopsis* reveals that auxin-inducible, but not repressible promoters, are enriched for specific composite *cis*-element modules. To analyse the distribution of AuxRE, ZRE and MRE *cis*-elements in all auxin-responsive promoters on a genome-wide scale, we made use of publicly available AtGenExpress *Arabidopsis* microarray datasets to initially identify auxin-regulated genes. The microarray data applied was part of an auxin time-course experiment with 7-days-old *Arabidopsis* Col-0 wt seedlings [43]. Samples from mock and 1 μM IAA (indole-3-acetic acid) treated plants were taken at 0.5, 1 and 3 h after treatment onset. After normalization, genes significantly 2-fold induced or repressed at the respective induction time points compared to the controls were identified to obtain a nearly exhaustive list of auxin-responsive genes. For nearly every regulated gene call, corresponding promoter sequences were assignable, giving the selected promoter dataset good coverage (Additional files 1,2). Subsequently the promoter sequences were grouped into 6 classes, taking into account whether their corresponding genes were up- or down-regulated after the different induction periods. The resulting groups were then scanned for the presence of specific AuxREs, ZREs and MREs (Table 1) and their significant enrichment or depletion was determined using the previously described real randomization algorithm. The *cis*-element list, which has already been used in the promoter *cis-*element analysis of the *GH3* promoters, was expanded for the genome-wide analysis by the RY motif (CATGCATG sequence), which is bound by ARF-like B3-type TFs [30], and a MYC2 TF binding site (CACATG), which strongly resembles a consensus G-box (CACGTG) that is bound by bZIPs [24,34].

Concerning module compositions, all possible motif combinations between members of the AuxRE-, ZRE- and MRE/MYC-motif classes were tested. In order to capture the majority of putatively functionally interlinked motifs, large- or narrow-spaced *cis*-element modules were assessed with a flexible spacing of maximal 100 bps between the individual motifs. The determination of this flexible window size was based on the observation that ZRE and MRE motifs frequently resided near AuxREs in auxin-responsive *GH3* promoters (Figure 1), which is consistent with previously published ZRE-AuxRE [15] and MRE-AuxRE [18] module discoveries. Moreover this spacing should cover the vast majority of postulated plant *cis*-element modules [44]. To take account of multiple testing errors that result from analysing several motifs and motif combinations, the actual p-values were corrected for all determined parameters by applying a Bonferroni correction factor. Only corrected p-values of an α ≤ 0.05 were retained.

**Figure 3:**
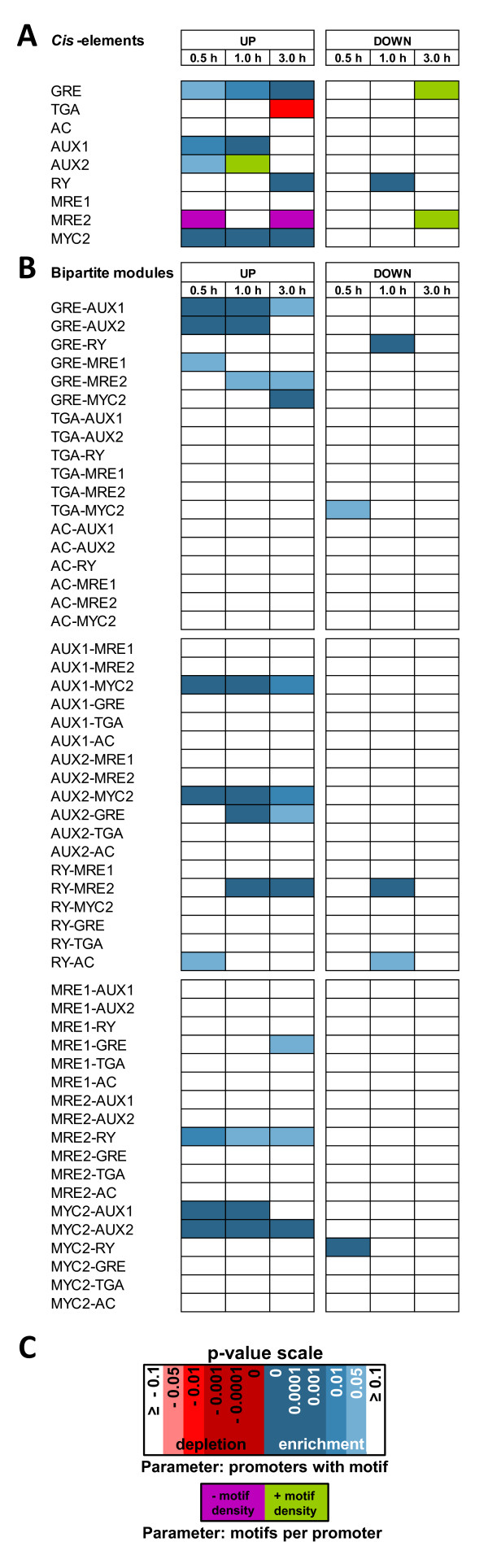
**Motif enrichment or depletion of specific *cis*-elements in auxin-responsive promoters from *A. thaliana.*** Statistical significant motif enrichment or depletion of specific ZRE, MRE/MYC and AuxRE related *cis*-elements (Table 1) in early (0.5 – 1 h post auxin treatment) and late (3 h post-treatment) auxin-responsive promoters from *Arabidopsis thaliana*. **A**) Motif enrichment or depletion for individual motifs in promoters of auxin-regulated genes. **B**) Significant enrichment or depletion of bipartite motif modules comprising ZRE, MRE/MYC and AuxRE *cis*-elements in promoters of auxin-regulated genes. The embedded motifs have a variable but maximal spacing of 100 bps. **C**) The significance level, which is defined by the determined Bonferroni corrected p-values, is displayed as colour-scale. Enriched motifs or modules with respect to parameter I are illustrated in shades of blue, whereas depleted motifs/modules are given in shades of red. Motif abundance that is significantly altered with respect to parameter II is coloured green for motif density enrichment and purple for motif density depletion.

Figure 3A - C illustrates the results from this genome-wide *cis-*element analysis. At first sight it became apparent that the analysed AuxRE, ZRE and MRE/MYC motifs and composite motif-modules were disproportionately more enriched in auxin-inducible rather than repressible promoters. A closer examination of the single enriched motifs revealed that, as expected, the typical ARF-like B3-type TF binding sites such as AUX1 and AUX2, but also the RY motif, were strongly enriched in the auxin-inducible promoters at early or late induction time-points. Regarding the bZIP-TF binding sites (ZREs), it was obvious that the single GRE motif as well as GRE-AuxRE associations occurred in significant, higher frequency (Figure 3A, B). Although the bipartite motif-module showed no orientation specificity to 5’ or 3’ positioning in general, the GRE-AuxRE module seemed to be preferred compared to the AuxRE-GRE module constellation (Figure 3B). Other ZREs, like the TGA- and AC motif, were per se and in combination with other motifs, generally not enriched or even depleted (Figure 3A, B), indicating that among all auxin up-regulated *Arabidopsis* promoters, the GRE motif seems to be the most preferred bZIP binding site.

Examining the abundance of the MREs it was noticeable that the single MRE2 *cis*-element was rather depleted in the auxin-regulated promoters. However, both analysed MRE motifs (MRE1 and MRE2) occurred at a significantly higher frequency in combination with the GRE motif (Figure 3B). Moreover, MRE2 associations with the RY B3-type TF binding site could be found.

Finally, the MYC2 motif (CACATG) was highly enriched as single motif or MYC2-AuxRE module in the promoters of auxin up-regulated genes (Figure 3B). As this site closely resembles a GRE (CACGTG), it remains to be disclosed which kind of TF binds in the context of auxin-induced transcription.

While Figure 3A, B illustrate which motifs or modules were enriched, they do not determine which of the tested motifs were most abundant in the analysed dataset. Therefore, all significantly enriched motifs (p ≤ 0.05) were additionally ordered with respect to the quantity of promoters in which they occurred. As it has been reported, in agreement with our results (Figure 1), that MREs and GREs reside in individual promoters near AuxREs [15,18] and, thus, might synergistically contribute to auxin-mediated transcription, we expanded the analysis to include triple motif modules of all three motif classes. An excerpt of the results which display the *cis*-elements which are present in at least 5% of the analysed promoters are presented as an occurrence list of single motifs and bi- or tripartite motif-module combinations at each time point (Table 2) and are summarized in the following remarks.

**Table 2:** **Occurrence list of enriched motifs and modules in early and late auxin-inducible promoters from *A. thaliana***

**combination**	**ORDER**	**0.5 h UP (179)**		**1 h UP (250)**		**3 h UP (398)**
**singles**	1	AUX2 (134)	1	GRE (172)	1	GRE (273)
	2	GRE (123)	2	MYC2 (136)	2	MYC2 (197)
	3	AUX1 (93)	3	AUX1 (129)	3	RY (27)
	4	MYC2 (91)		..		..
		..	1*	AUX2 (181)		..
**bipartite**	1	GRE-AUX2 (33)	1	GRE-MRE2 (122)	1	GRE-MRE2 (188)
	2	GRE-MRE1 (32)	2	GRE-AUX2 (43)	2	MRE1-GRE (65)
	3	MYC2-AUX2 (27)	3	AUX2-GRE (39)	3	MYC2-AUX2 (42)
	4	AUX2-MYC2 (23)	4	MYC2-AUX2 (34)	4	GRE-MYC2 (37)
	5	GRE-AUX1 (21)	5	GRE-AUX1 (32)	5	AUX2-MYC2 (35)
	6	AUX1-MYC2 (14)	6	AUX2-MYC2 (28)	6	GRE-AUX1 (31)
	7	MYC2-AUX1 (13)	7	AUX1-MYC2 (17)	7	AUX1-MYC2 (20)
	8	MRE2-RY (9)	8/9	RY-MRE2 (13)	8	RY-MRE2 (18)
		..	8/9	MYC2-AUX1 (13)	9	MRE2-RY (15)
		..	10	MRE2-RY (11)		..
**tripartite**	1	GRE-AUX2-MRE2 (22)	1	GRE-AUX2-MRE2 (27)		..
	2	GRE-AUX1-MRE2 (15)	2	GRE-AUX1-MRE2 (20)		..
	3	MRE2-GRE-AUX1 (13)	3	MRE2-GRE-AUX1 (19)		..
	4	GRE-AUX2-MRE1 (8)	4	GRE-AUX2-MRE1 (9)		..

Enrichment of *cis*-elements in the promoters of significantly (2-fold) auxin-induced genes determined by parameter I. The analysis discriminates between early (0.5 – 1 h) or late (3 h) responding genes. Asterisks indicate enrichment with respect to parameter II. The number of analysed promoters as well as that of promoters containing at least one of the presented motifs or modules is given in parentheses. An extended list of enriched motifs which also considers *cis*-elements which are present in less than 5% of the analysed promoters can be found in Additional file 3. Presented modules (bipartite, tripartite) exhibit a variable but maximal spacing of 100 bps between each embedded motif. Sequences and abbreviations of *cis*-elements are given in Table 1.

#### Single motifs

The well-characterized AuxREs (AUX1 and AUX2) and the relatively rare RY motif (within 3% of the *Arabidopsis* promoters) were highly enriched in early or late auxin-inducible promoters, respectively. Besides these, the GRE and its related MYC2 motif were listed in the set of early and late auxin-inducible promoters among the top 3 most abundant *cis-*elements. The TGA motif, which has been shown to be frequently present in the promoters of the early auxin-responsive *GmGH3* homologs, was slightly depleted as a single motif within the set of all *Arabidopsis* auxin-inducible promoters in this analysis. However, it should be considered that this depletion was calculated on the basis of parameter I (“number of promoters with a motif”) compared to a randomized background dataset. In fact, several promoters within the dataset of auxin-inducible genes still contained this motif.

#### Bipartite motifs

GRE-AuxRE associations were the predominantly enriched bipartite motif combination within the group of early (0.5 h after IAA exposure) auxin-inducible promoters. However, this association was also highly abundant in promoters that responded 1 h or 3 h after auxin treatment. Furthermore, it was noticeable that MYC2-AuxRE *cis*-regulatory units were generally enriched in early to late, and GRE-MRE modules in rather late, auxin-inducible promoters. Consistent with the observation that slight variations of the consensus AuxRE (AUX1) have been commonly found in auxin-responsive promoters [17,45] the majority of GRE-AuxRE and MYC2-AuxRE combinations were not associated with AUX1 but to AUX2.

#### Tripartite motifs

Examining the distribution of tripartite modules of ZRE, MRE/MYC2 and AuxRE motifs, it was apparent that GRE-AUX1/2-MRE1/2 combinations could be found in early auxin responsive promoters (0.5 - 1 h post treatment). Notably, in all of these modules the GRE motif resided next to an AuxRE, whereas the position of the MRE motif in this module seemed to be more variable. However, in 80% of the GRE, AuxRE and MRE tripartite modules, the MRE motif also resided near an AuxRE.

By summing up these results it can be pointed out that the auxin-inducible promoters revealed an enrichment of single AUX1/2, GRE, MYC2 and RY *cis*-elements. Moreover, in particular GRE, MYC2 and MRE motifs were frequently associated with AuxREs.

### *Cis*-elements and modules of AuxREs, ZREs and/or MRE/MYCs are also enriched in auxin-inducible promoters from rice

**Figure 4:**
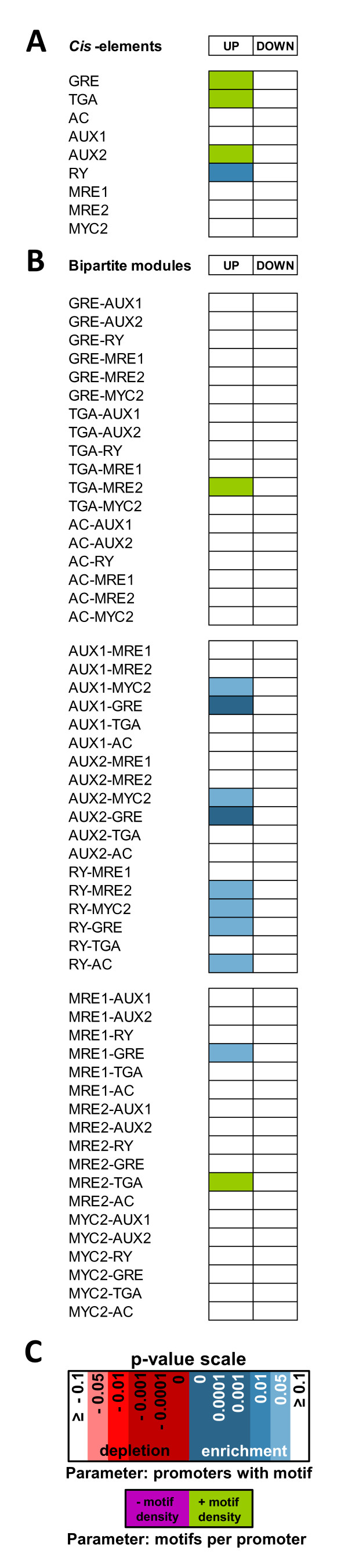
**Motif enrichment or depletion of specific *cis*-elements in auxin-responsive promoters from rice.** Statistical significance of motif enrichment or depletion for specific ZRE, MRE/MYC and AuxRE related *cis*-elements in auxin-responsive promoters from *Oryza sativa*, 1–3 h after auxin treatment. **A**) Motif enrichment or depletion for individual motifs in promoters of auxin-regulated genes. **B**) Significant enrichment or depletion of bipartite motif modules comprising ZRE, MRE/MYC and AuxRE *cis*-elements in promoters of auxin-regulated genes. The embedded motifs have a variable, but maximal spacing of 100 bps. **C**) The significance level scale for the Bonferroni corrected p-values of parameters I and II is adapted from Figure 3.

To assess whether the *cis*-element distribution observed in the *Arabidopsis* auxin-inducible promoters is evolutionary conserved, we analysed a publicly available microarray dataset from the monocot plant *Oryza sativa*. In the corresponding work [46], transcript preparations from 7-days-old rice seedlings treated with auxin for 1 and 3 h were pooled and compared to those of non-induced plants. Since the experiment contained no induction time series, only up- or down-regulated genes could be identified. After normalization, these genes were classified with respect to a significant 2-fold expression difference, resulting in the identification of 203 up- and 79 down-regulated genes (Additional files 2,4). The compiled promoter lists were subsequently analysed for single motifs and double and triple motif combinations as it has been done for the *Arabidopsis* dataset (Figure 4A – C; Table 3). This analysis revealed, that auxin-inducible rice promoters exhibited an enrichment of ZRE-, MRE- or AuxRE-motifs and composite modules, rather than those which were repressed after the hormone stimulus. More precisely, not only the single AUX2 and RY elements were significantly enriched, but also the GRE and TGA motifs; however some of them only with respect to enhanced motif density. Concerning the bipartite modules, it was similar to the *Arabidopsis* auxin-inducible promoters that GRE and MYC2 associations with ARF-like B3-type TF binding sites (AUX1 or AUX2) were highly enriched. In contrast to results obtained with *Arabidopsis* though, composite TGA-MRE2 modules were overrepresented in rice. These results are also reflected in the ordered promoter lists in which the enriched motifs and modules that at least appear once in ~ 5% of the analysed promoters were sorted according to the number of promoters in which they occur (Table 3). In this list, the single RY element outranked all other motifs followed by the GRE-, AUX2 and TGA *cis*-elements that were enriched with respect to parameter II. Referring to the bipartite motif modules, the AuxRE-GRE, MRE-GRE and AuxRE-MYC2 combinations were highly abundant. Within the group of complex tripartite modules, GRE, AuxRE and MRE2 containing modules were most preferred, although TGA, AuxRE, MRE module combinations were also enriched, in which the TGA motif substituted the GRE *cis*-element.

**Table 3:** **Occurrence list of enriched motifs and modules within auxin-inducible promoters from *O. sativa***

**combination**	**ORDER**	**UP (203)**
**singles**	1	RY (26)
	1*	GRE (144)
	2*	AUX2 (140)
	3*	TGA (130)
**bipartite**	1	AUX2-GRE (45)
	2	MRE1-GRE (39)
	3	AUX2-MYC2 (26)
	4	AUX1-GRE (21)
	5	RY-MRE2 (14)
	6	AUX1-MYC2 (12)
	1*	TGA-MRE2 (89)
	2*	MRE2-TGA (83)
**tripartite**	1	AUX2-GRE-MRE2 (25)
	2	MRE2-AUX2-GRE (24)
	3	AUX1-GRE-MRE2 (13)
	4/5	MRE2-AUX1-GRE (12)
	4/5	AUX1-MRE2-TGA (12)

Enrichment of *cis*-elements in promoters of significantly (2-fold) auxin-induced genes was determined by parameter I, whereas asterisks indicate an enrichment with respect to parameter II. The number of analysed promoters as well as that of promoters containing at least one of the presented motifs or modules is given in parentheses. An extended list which also includes more rare motifs and modules can be found in Additional file 3. Presented modules (bipartite, tripartite) exhibit a variable, but maximal spacing of 100 bps between each embedded motif. Sequences and abbreviations of *cis*-elements are given in Table 1.

### Comparative analysis of enriched *cis*-elements and modules from *Arabidopsis* and rice displays a conservation of specific *cis*-regulatory elements

In order to examine whether specific motifs or composite modules are conserved in auxin-induced promoters of *Arabidopsis* and rice, we performed a comparative analysis of the promoter occurrence of each enriched *cis*-regulatory element. Unfortunately, the available microarray data from the auxin-induction experiments from *Arabidopsis* and rice were differently designed so that comparable datasets had to be determined. As the number of genes which were not induced after 1 h but explicitly after 3 h of auxin application was rather small in the *Arabidopsis* dataset, we decided to compare the dataset of the 1 h up-regulated genes from *Arabidopsis* to that of the combined 1 to 3 h dataset from rice (Tables 2, 3). Although we cannot exclude that rice and *Arabidopsis* plants respond differently to the auxin application, a comparison of these datasets resulted in a largely concurrent set of enriched motifs in both analysed plant species.

This comparison revealed that the enrichment of single AUX2, GRE and RY motifs within the promoters of auxin up-regulated genes is evolutionary conserved between the dicot and the monocot model plant. Considering the bipartite motif combinations, several modules that were highly enriched in the *Arabidopsis* dataset were also present in the ordered promoter occurrence list of rice. Most noticeable were GRE, AuxRE containing modules as well as GRE, MRE1/2 and MYC2, AUX2 motif combinations, that appeared at various positions in the occurrence lists of both angiosperm plant species. With respect to the tripartite modules, the GRE-AuxRE-MRE2 or its related MRE2-AuxRE-GRE module were found to be enriched in *Arabidopsis* or rice. Although even more module combinations of these three binding sites were significantly overrepresented in auxin-inducible promoters from both species, it was striking that in all of them the GRE motif was located near an AuxRE.

Besides these similarities the most distinct difference in terms of motif enrichment related to the TGA *cis*-element. It was found to be frequently abundant in the promoters of *GmGH3* homologs (Figure 1) and was occasionally enriched as single motif and in composite bi- and tripartite-motif modules with MREs and/or AuxREs in the promoters of auxin up-regulated genes from rice. In contrast, it was not overrepresented in the auxin-responsive promoters from *Arabidopsis.*

### Distinct motifs and composite *cis*-regulatory modules are enriched within promoters of auxin-induced *Arabidopsis* gene families

To create a more detailed profile which auxin-responsive genes might actually be regulated by which specific type of motif or module, we analysed the promoters of well-characterised auxin-regulated gene families such as the *GH3sAUX/IAAs* and *SAURs* which are described to be early auxin-responsive [17,47] (Additional file 5). However, all of them also include some late responsive members [48]. In addition, the family of the auxin-related *ARF* genes was studied as an example of an auxin-related, but largely constitutively expressed gene group [6] (Additional file 5). In the following section an overview of the results from this gene family specific *cis*-element analysis is given (Table 4).

**Table 4:** **Occurrence list of enriched *cis*-elements and modules in promoters of auxin-responsive gene families from *A. thaliana***

**combination**	**ORDER**	**AtGH3 (20)**		**AtAUX/IAAs (29)**	**AtARFs (23)**		**AtSAURS (80)**
**singles**	1	MYC2 (13)	1	MYC2 (18)	..	1	MYC2 (47)
		..	1*	AUX2 (21)	..	1*	AUX2 (58)
		..	2*	AUX1 (17)	..		..
**bipartite**	1	MYC2-AUX2 (5)	1	MYC2-GRE (7)	..	1	MYC2-AUX2 (17)
	2	MYC2-AUX1 (3)	2	AUX2-MYC2 (6)	..	2	AUX2-MYC2 (14)
		..	3/4	AUX1-MYC2 (5)	..	3	AUX1-MYC2 (9)
		..	3/4	MYC2-AUX2 (5)	..		..
**tripartite**	1/2	GRE-AUX2-MRE1 (3)	1	MRE2-AUX2-GRE (6)	..		..
	1/2	AUX2-MRE1-GRE (3)	2	MRE1-AUX2-GRE (3)	..		..
	3-10	AUX2-TGA-MRE1 (2)	3-6	MRE1-GRE-AUX1 (2)	..		..
	3-10	GRE-MRE1-AUX2 (2)	3-6	MRE1-AUX1-GRE (2)	..		..
	3-10	GRE-MYC2-AUX1 (2)	3-6	AUX1-MYC2-GRE (2)	..		..
	3-10	GRE-MYC2-AUX2 (2)	3-6	AUX2-MYC2-GRE (2)	..		..
	3-10	MYC2-AUX2-TGA (2)		..	..		..
	3-10	MYC2-AUX2-GRE (2)		..	..		..
	3-10	GRE-AUX1-MRE1 (2)		..	..		..
	3-10	AUX1-MRE1-GRE (2)		..	..		..

Given is the significant motif and module enrichment or depletion in the promoters of auxin-related gene families regarding parameter I, whereas asterisks indicate an enrichment with respect to parameter II. The number of analysed promoters as well as that of promoters containing at least one of the presented motifs or modules is given in parentheses. An extended list which takes account of more rare motifs (occurrence in < 5% of analysed promoters) can be found in Additional file 3. Presented modules exhibit a variable, but maximal spacing of 100 bps between each embedded motif. Sequences and abbreviations of *cis*-elements are given in Table 1.

*GH3s –* The promoters of the *Arabidopsis GH3* gene family were enriched for the single MYC2 motif and bipartite MYC2-AuxRE modules. In the group of tripartite modules, the GRE-AUX2-MRE1 was enriched and outranked other motif combinations. Although TGA motifs were found to be depleted in the set of all auxin-induced promoters (Figure 3, Table 2), they were significantly enriched as tripartite AUX2-TGA-MRE1 or MYC2-AUX2-TGA modules within the *Arabidopsis GH3* promoters.

*Aux/IAAs –* The single MYC2 and AuxRE *cis*-elements were significantly overabundant in the *Arabidopsis AUX/IAA* promoters. Moreover, modules of MYC2-AuxRE and MYC2-GRE were found with higher frequency. Regarding the complex tripartite modules, several constellations of MRE1/2-AuxRE-GRE associations were most prominent.

*SAURs –* In *Arabidopsis*, the promoters of the *SAUR* gene family were significantly enriched for the single MYC2 and AUX2 motifs. This conservation also persisted in the bipartite modules of MYC2 and AuxREs.

*ARFs –* The promoters of the *ARF* gene family displayed no significant enrichment for the *cis*-elements and modules that were analysed. Obviously the transcriptional control of this, in general constitutively expressed gene family [6], is not dependent on the presence of these *cis*-regulatory elements.

In conclusion, it could be demonstrated that the analysed auxin-regulated gene families from *Arabidopsis* showed in part distinct, but also common *cis*-regulatory elements (Table 4). Noticeable in this respect was the observation that the primarily early auxin-responsive gene families, such as the *GH3s, Aux/IAAs* and *SAURs* exhibited an enrichment of the single MYC2 as well as MYC2-AuxRE modules, which were missing in the promoters of the mainly, constitutively expressed *ARF* genes. Furthermore, it has to be noted that GRE, AuxRE and MRE1/2 containing tripartite modules were frequently enriched in the promoters of the *GH3* and *AUX/IAA* gene families, but not in the *SAURs* or *ARFs*. Taken together, these observations suggest a certain dependency of specific *cis*-elements in the regulation of distinct auxin-regulated gene families and early auxin-responsive gene classes.

### Mutations in the GRE *cis*-elements within a GRE-AuxRE module of the *Arabidopsis GH3.3* promoter lead to a significant reduction of its auxin-responsiveness

**Figure 5:**
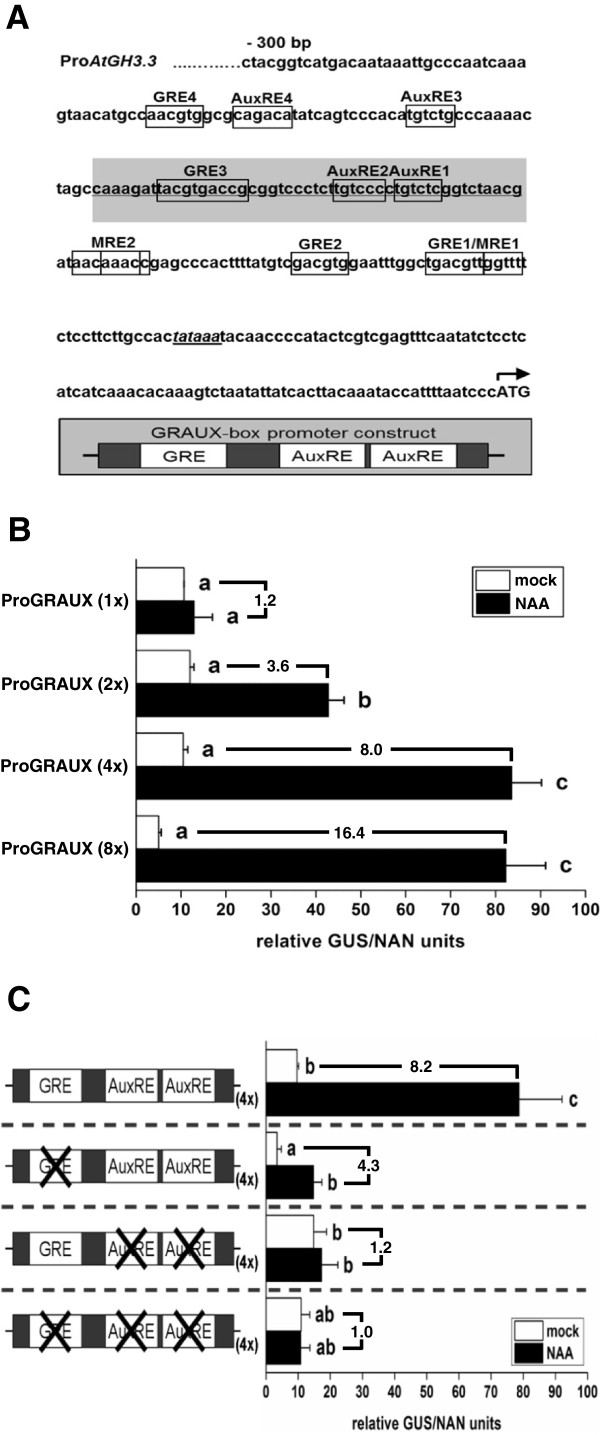
**Molecular characterisation of the GRAUX-module. A**) GRE, MRE and AuxRE *cis*-elements within the −300 bp *AtGH3.3* promoter region. Framed sequences indicate the positions of the GRE-, MRE- and AuxRE motifs within the *AtGH3.3* promoter close to the transcriptional start site (TATA-box is underlined). The present *cis*-elements are serially numbered. The grey highlighted sequence represents the *AtGH3.3* promoter region used as synthetic auxin-responsive GRAUX-module promoter construct. **B**) Expression profile of the synthetic ProGRAUX: GUS reporter constructs. Number of multimerisations and fold induction values are indicated. **C**) Auxin inducibility of the ProAtGH3.3 derived GRAUX(4x)-module reporter construct and its mutational derivates. A schematic view of the transfected reporter constructs is given. Mutated *cis*-elements are indicated by X. White coloured bars represent transfected, mock treated (DMSO) and black coloured bars NAA treated (0.25 μM, 16 h) samples. Presented results were obtained from transient protoplast transfection assays. Given are the mean GUS/NAN values (± SD) from 3 independent experiments. Different letters denote significant differences (p ≤ 0.05; one-way ANOVA followed by Fisher post-hoc test) between the used constructs and treatments. Fold induction values resulting from auxin application are given

The previously described *cis*-element analyses repeatedly indicated that particularly GRE-AuxRE modules are highly enriched and conserved in auxin-inducible promoters. In order to validate the bioinformatic based assumption that GRE and AuxRE *cis*-elements co-operate in auxin-mediated transcription, we inspected the promoters of the early auxin-responsive *Arabidopsis GH3* genes for the presence of GREs. *AtGH3.3* is a close homolog of the well-characterised soybean *GmGH3* and harbours several GREs in its promoter in close vicinity to AuxRE- and MRE *cis*-elements and to the transcriptional start site (TSS) (Figure 5A, Figure 1). In fact, some of them also form the GRE-AuxRE-MRE tripartite module, which was shown to be frequently enriched in *AtGH3* promoters (Figure 5A, Table 4).

To analyse the effect of the GRE motif on auxin-mediated transcription, a short synthetic *AtGH3.3* derived promoter region containing one GRE and two AuxRE *cis*-elements was constructed which we termed GRAUX module (GRE-AuxRE, Figure 5A). Multimers of this module were fused to a minimal promoter and the GUS reporter gene, allowing expression analysis by transient protoplast transfection assays. Whereas a single GRAUX module construct exhibited a slightly, but not significantly higher reporter gene expression in the presence of low exogenous auxin concentrations, multimerisation strongly enhanced auxin-responsiveness (Figure 5B). Notably, all constructs showed a similar background expression under non-inductive conditions (Figure 5B).

To assign the influence of the GRE motif within the GRAUX module, *cis*-element specific mutational derivatives of the 4-times multimerised construct were analysed. By this means, it was demonstrated that mutations in the core sequence of the GRE-motif resulted in a ~ 50% reduction of the auxin-triggered inducibility, while mutations in the AuxREs alone or in combination with a mutated GRE led to complete auxin insensitivity (Figure 5C). As we have demonstrated that auxin-inducible promoters are in general enriched for GRE-AuxRE modules, this functional analysis underpins that they are certainly potential quantitative elements in auxin-regulated promoters.

## Discussion

In this work, we have presented a genome-wide *cis*-element analysis of bZIP- (ZRE), MYB/MYC- (MRE/MYC2) and ARF-TF (AuxRE/RY) related binding sites in auxin-responsive promoters from *Arabidopsis* and rice. We could demonstrate that specific *cis*-elements and/or composite modules, which encompass typical binding sites for these TFs, are enriched in auxin-inducible promoters of the tested angiosperm plant species. Most prominent and concurrent in this respect, is the enrichment of the single GRE and AUX1/2 motifs as well as their related bi- and tripartite-module organisations, in which they are also commonly associated with MREs. Moreover, a substantial enrichment of the relatively rare RY *cis*-element and of MYC2-AuxRE modules was observed. The evolutionary conservation of these *cis*-acting elements in the analysed monocot and dicot model plants, that exhibit considerable differences in their species specific genome structure in terms of the GC content, affirms that they might be part of a common regulatory mechanism in auxin-responsive transcription. Ensuing studies are of importance to define the relevance and function of the identified motifs and to disclose which sets of auxin responsive genes are regulated by them. In our work a detailed gene family specific *cis*-element analysis revealed that especially promoters from early auxin-responsive gene classes are enriched for bipartite MYC2-AuxRE and tripartite GRE-AuxRE-MRE modules. In fact, we demonstrated that mutations in the GRE motifs within a promoter construct derived from the early auxin-responsive *AtGH3.3* gene result in a severe reduction of its auxin-triggered inducibility.

### The applied randomization algorithm determines motif enrichment to predict the functional relevance of regulatory *cis*-elements

In the presented *cis*-element analyses, we focused on the enrichment and distribution of specific motifs in auxin-responsive promoters to predict their *cis*-regulatory importance. However, the validity of the bioinformatically founded hypotheses is based on and limited by the main assumption that all or a relevant proportion of target genes, that are regulated by the same type of TF, should contain the TFs’ cognate binding site in their promoters. This is certainly not mandatory as individual TFs might co-operatively regulate their target genes by interacting with other DNA-bound transcriptional regulators [49]. Besides this it has to be considered, that inherent algorithm limitations can negatively affect p-value determination for motif enrichment or depletion. In this study almost all analysed *cis*-elements were normally (or near normally) distributed in the background population, generated by the applied randomization algorithm. Hence, accurate p-values for determining motif enrichment could be calculated using Gaussian Z-statistics. However, extremely rare and extremely overabundant motifs (e.g. MRE2) follow different distributions (most likely Poisson or Hypergeometric ones) as they are non-normally distributed. This hampers reliable distribution analysis as the p-values delivered by the randomization algorithm become inaccurate. Therefore we provide additional significance parameters such as parameter II (“motif density”) to take account of this limitation. Despite these general restrictions, we have demonstrated that the employed Motif Mapper *cis*-element analysis software and the integrated randomization algorithm proved to be straight-forward and effective for accessing the significance of the tested *cis*-element and composite *cis*-element module distributions. In contrast to other more complex *cis*-regulatory module scanners that require clusters of tightly co-expressed genes and/or sets of orthologous genes [38,50], the algorithm performed well in noisy, unclustered datasets. This is possible as the algorithm simply defines the number of motifs and/or modules in the genomic, randomized and experimental datasets to determine motif enrichment or depletion without seeking for optimal concurrent *cis*-elements to explain a given dataset clustering in its entity. Therefore, any association of genes can be chosen (e.g. gene families, GO annotations, etc.) and analysed for any type of *cis*-element or module distribution. The user only needs to provide a complete, genomic set of promoters of which several are now available, a specific promoter subset of interest and a list of normally distributed *cis*-elements and/or motif combinations that should be tested.

### ZRE and MRE *cis*-elements are potential quantitative AuxRE coupling elements

Specific ZRE, MRE/MYC2 and AuxRE-related motifs and composite modules were found to be significantly enriched in the auxin-responsive promoters from *Arabidopsis* and rice (Figures 2,3; Tables 2,3). The most outstanding in this respect are the single AuxRE, GRE- and the relatively rare RY-motifs, which were enriched in the promoters from all auxin up-regulated genes from *Arabidopsis* and rice, followed by the MYC2 element, which was enriched in the *Arabidopsis* auxin up-regulated promoters and the MRE2, which was most frequent in the auxin-inducible promoters from rice. However, the relevance of some of these motifs in terms of enrichment was more pronounced and of others, only concrete in association with additional motifs. Particularly, the MRE motifs were frequently enriched in a modular structure with AuxREs and/or GREs in the auxin-inducible promoters from both analysed species (Figures 2,3; Tables 2,3). Thereby, the MRE2 seemed to be preferred compared to MRE1, whereas this could be partially explained by the fact that the MRE1 motif is slightly rarer than MRE2. The observation that MREs frequently resided near AuxREs (Figure 1), especially in tripartite GRE-AuxRE-MRE motif combinations (Tables 2,3), suggests that MREs are a relevant integration platform for MYB-TF activities in auxin-mediated transcription. In fact, this assumption is supported by experimental data provided by Shin and co-workers [18]. Consistent with our findings, they were able to show that AtMYB77 effectively interacts with AtARF7 and other ARFs to synergistically promote target gene expression [18]. This indicates that MREs likely function as coupling elements for AuxRE-mediated transcription in both, monocot and dicot plant species.

Similar observations can be made for specific ZRE motifs. Some of them occurred in high frequency in the context with adjacent AuxRE elements. In this respect, the bipartite GRE-AuxRE or tripartite GRE-AuxRE-MRE2 modules were the by far most dominant combinations in early auxin-inducible promoters from both species (Figures 2, 3; Tables 2, 3). In particular the tripartite module was found to be enriched in early auxin responsive gene families, such as the *GH3s* or *AUX/IAAs* (Tables 4). In fact, the experimental validation of the functional relevance of the GRE motif as quantitative AuxRE coupling element by mutational *cis*-element analysis revealed that it at least significantly contributes to the auxin-mediated induction of the *AtGH3.3* gene (Figure 5 A- C).

Consistently, it has been reported that G-box like motifs are associated with AuxREs in the early auxin-responsive promoters of the soybean *GH3* and *AUX28* genes and that they synergistically promote transcription [15,19-21]. Furthermore, it was demonstrated that a tobacco *NtGH3* gene harbours multiple GRE-AuxRE repetitions in its promoter and that the bZIP factors NtBZI-1 and NtBZI-2, which had been implicated to regulate specific auxin responses, bind to them [22,51]. The homologous *Arabidopsis* group C/S1 bZIP transcription factors have been characterized to be involved in reprogramming transcription in response to low energy stress [52,53]. Therefore, it is tempting to speculate that these TFs might integrate information about the cellular energy homeostasis into auxin-specific expression patterns.

### MYC2 and RY binding sites are strongly enriched *cis*-elements in auxin-responsive promoters with yet undefined relevance

Based on the *cis*-element analyses of auxin-responsive promoters from *Arabidopsis* and *rice* we detected a significant enrichment of MYC2 and RY *cis*-elements in auxin-inducible promoters (Figures 2,3). Concerning the MYC2 motif, we observed a strong enrichment of the single and bipartite MYC2-AuxRE module in the auxin-inducible promoters from *Arabidopsis* and more precisely, in the promoters of the early auxin-responsive *AtGH3AtAUX/IAA* and *AtSAUR* gene families (Table 4). Consistent with this, Pufky and co-workers [54] revealed in an unbiased approach that MYC2 motifs were overrepresented in small clusters of auxin-inducible promoters from *Arabidopsis* compared to their expected genomic frequency. Moreover, Nemhauser and co-workers [50] described that MYC2-related *cis*-elements are overabundant in promoters of auxin- and brassinosteroid- responsive genes. Even though these findings suggest that the MYC2 motif might be involved in modulating the expression of auxin-responsive genes, its function is yet unclear. Recently, it was reported that the MYC2-motif is enriched in promoters from diurnally regulated genes and that it is sufficient to confer the observed circadian expression pattern in vivo [55]. As many growth related processes, which are mainly mediated by auxin, are also interconnected with the circadian clock [56], the MYC2-element might provide a molecular link between these systems. However, as the MYC2- (CACGTG) and the GRE (CACATG) motif share high sequence similarity, it has to be addressed which type of TF actually binds the promoters in this context.

Regarding the single RY motif, we have demonstrated that it is enriched in auxin-inducible promoters from *Arabidopsis* and rice, even though it was only present in 3% of all *Arabidopsis* and 7% of all rice promoters. The RY motif or Sph/Ry-box [57] was reported to be involved in abscisic acid (ABA) signalling [58]. It is known to be bound by members of the B3-type TF superfamily, which also includes ARF TFs. One of these members, which directly binds the RY *cis*-element, is ABI3, which contains three basic domains, originally designated B1, B2 and B3 [57]. Whereas the B3 domain was demonstrated to be necessary for RY binding [57], the B2 domain appears to be responsible for recruiting bZIP TFs [59-61]. An example of such interplay between ABI3 and bZIP TFs was described by Alonso and co-workers [49], which reported that these TFs synergistically promote the expression of seed maturation genes, including seed storage and dehydration-responsive genes. Based on these observations it is conceivable that RY binding TFs might functionally interact with bZIPs in auxin-responsive gene expression.

## Conclusion

In order to adjust auxin-controlled growth and developmental processes in plants in response to the diversity of fluctuating environmental cues, integrative *cis*-regulatory elements are needed to quantitatively modulate auxin-dependent gene expression, which is mediated by the well-characterised qualitative AuxRE motif. Indeed, modular organisations of GRE- and MRE-motifs in association with AuxREs were found to be highly enriched and evolutionary conserved and appeared to synergistically contribute to auxin-inducible expression. The finding that MYC2 and RY motifs were highly enriched in auxin-responsive promoters, further increases the combinatorial, integrative opportunities in auxin-mediated transcription. These results demonstrate the potential of bioinformatic approaches to generate working hypotheses on putatively relevant *cis*-regulatory elements in order to design experiments to characterize their predicted functions.

## Methods

### Phylogenetic and *cis*-element analysis of *GH3* promoters

*GH3* promoter sequences were sourced from the Plant Genome Database [62]. All available plant genomes were queried and scanned for putative *GmGH3* [NCIB: accession X60033]; [23] homologs using tBLASTX. Sequence matches with BLAST scores ≤ 1x10^-160^ were retained to identify putative orthologs. Promoter sequences (−1000 bps) were extracted from plant genomes with sufficient sequence coverage. AuxREs, ZREs and MREs were mapped and illustrated using TOUCAN 2 [39]. The GH3 protein sequences which were obtained from their corresponding cDNA sequences were used for subsequent phylogenetic analysis applying the ClustalW2 software at the EMBL-EBI sever using the default parameters [63]. The phylogenetic tree, rooted to *Pp*GH3-1 was rendered with TreeView software [64].

### Microarray analysis

Auxin-responsive genes from *A. thaliana* and *O. sativa* were identified by analysing the microarray data from auxin treatment experiments. For *Arabidopsis*, a wild type seedling experiment with an induction time-course of 1 μM IAA for 30 min, 1 h and 3 h [TAIR ExpressionSet: 1007965859] was used and for rice (*Oryza sativa* variety IR64) a wild type seedling experiment with pooled transcript samples from plants that were induced by auxin for 1 and 3 h, respectively (NCBI Gene Expression Omnibus GSE5167). The corresponding data files were imported into GenSpring GX 7.3.1 (Agilent) and the datasets adjusted to remove the background of optical noise using the GC-RMA software [65]. After quantile normalization the R LIMMA package [66] for GC-RMA was applied to identify significant gene-expression changes compared to the control samples. In downstream analyses, only genes significantly 2-fold up- or down-regulated (p ≤ 0.05) after auxin treatment compared to controls were used.

### Promoter sequence retrieval

The genomic *Arabidopsis* promoter dataset (‘TAIR9_upstream_1000_20090619’) was obtained from *Arabidopsis* TAIR 9 release [67] and cleaned from promoters of plastidial and alternative transcribed genes. *O. sativa* promoters (−1000 bps) were extracted from GenBank RAP BUILD 3 files provided by NCBI [68] using the GenBank extraction script aGBSQL of the Motif Mapper for Python v1.4 software [37] and choosing option root = 2 (take most 5’ annotation point for each gene [option TSS or ATG]). Promoters for the specific and randomized datasets were taken automatically from these FASTA formatted data files.

### Gene descriptions

Functional gene descriptions for *A. thaliana* were taken from TAIR9_functional_descriptions dump file. This list was cross referenced using the TAIR Gene Descriptions web based query tool and recent literature on auxin-related gene family classifications [36,69,70]. Redundant entries due to alternatively spliced transcripts were manually removed.

### Real randomization algorithm

The Cluster Analysis Real Randomization algorithm (Motif Mapper v5.2.4.01) was developed to facilitate the determination of *cis*-element and *cis*-element module distributions in a selected experimental dataset (n ≥ 2) and to define significant distribution alterations compared to at least 1000 randomly composed, equally sized, reference promoter datasets. These randomized datasets are compiled of promoters which are drawn from large genomic promoter datasets (e.g. from the *Arabidopsis* genome which has ~30000 promoters or Rice with ~60000) and are therefore highly variable. If complexity of the reference pools decrease (i.e. if significantly smaller species specific promoter datasets are used) we recommend increasing the number of analysed random datasets to 3000 – 5000. To determine motif enrichment or depletion, the algorithm initially extracts promoter sequences from the provided FASTA formatted input data file to create the experimental and randomized reference datasets. Afterwards, a list of defined motifs or modules is entered manually or per text file and the algorithm automatically maps them for the entire dataset. Motifs and composite modules can be analyzed on Watson strands only or include their Crick strand in the calculation (“auto-antisense”). Motifs can be of any size and modules composed of any number of motifs with any flexible spacing between them. In the presented study, modules were defined as multi-element *cis*-regulatory units that embedded motifs (Watson and Crick words; “dyadic-(auto)”) are not overlapping, but have a flexible spacing of maximal 100 bps between each other. The window size is based on the vast majority of yet published functional module descriptions [44]. After determination of motif (word-matching in the nucleotide string) or module (word-matching separated by flexible gaps) abundance, enrichment or depletion in the experimental dataset compared to the randomized background population is calculated using a standard Z-score. If needed, the obtained p-value scores and the output randomization data can be corrected by additional statistical tests. The results from the uncorrected and corrected p-value calculation for the four parameters: “number of promoters with a motif”, “number of motifs per promoter”, “the total number of motifs” and “the motif variance” are returned to the user per input promoter dataset file. To calculate the false-positive error rate; a script was written to reiteratively call the Cluster Analysis Real Randomization algorithm to determine motif enrichment for various sample sizes and motifs and is available upon request. The Motif Mapper software and instruction is available at [37] and descriptions about the Module Master algorithm at [71].

### Plant cultivation, protoplast transformation and GUS-assays

4 to 5 weeks-old *Arabidopsis* Col-0 plants grown on soil under long day conditions at 23 °C and a relative humidity of 60% were used for protoplast preparation. Protoplast isolation and transformation was performed according to Ehlert and co-workers [72]. For promoter activation assays 14 μg of the promoter: GUS reporter plasmid was co-transfected with 3 μg of a normalization plasmid (Pro35S: NAN). After transformation the protoplasts were incubated for 16 h in incubation buffer supplemented with 0.25 μM NAA (Sigma-Aldrich, Germany) or DMSO (mock). GUS and NAN enzyme assays were performed according to Kirby and Kavanagh [73]. The ratio of GUS to NAN activities is calculated as relative GUS/NAN values. Statistical analysis was performed using the software OriginPro 8.1 G (OriginLabs).

### Molecular biological techniques

Molecular Biology techniques have been described by Sambrook and co-workers [74]. DNA mutagenesis was performed applying the Quick Change site directed mutagenesis kit (Stratagene, Amsterdam, Netherlands) following the manufacturers’ instructions. DNA sequence analysis was performed using an ABI310 sequencer (Applied Biosystems, Darmstadt, Germany) with an ABI PRISM BigDye terminator cycle sequencing reaction kit (Applied Biosystems, Darmstadt, Germany).

### Design of promoter: GUS reporter constructs

The synthetic GRAUX-module promoter: GUS construct was designed using complementary oligo-nucleotides for the GRAUX-module sequence with a 5` flanking *Eco*RI and *Bcu*I and a 3` flanking *Xba*I restriction site. After hybridisation, the oligohybrid and the pBT10-GUS vector (B. Weisshaar, University of Bielefeld, Germany) were digested with *Eco*RI and *Xba*I and then ligated. Due to the additional 5` located *Bcu*I restriction site in the inserted GRAUX-module and the fact that *Bcu*I and *Xba*I digested fragments have compatible, cohesive ends, additional GRAUX-module fragments could be successively inserted leading to multimerised GRAUX-module promoters. A detailed description of the procedure can be found in [75]. The oligo sequences used for GRAUX-module construct design are: GRAUX-module_for_: 5`-AAAATTCACTAGTCAAAGATTACGTGACCGCGTCCCTCTTGTCCCCT GTCTCGGTCTAACGT-3` and GRAUX-module_rev_: 5`-CTAGACGTTAGACCGAGACAG GGGACAA GAGGGACCGCGGTCACGTAATCTTTGACTAGTG-3`. Existing GRE- and AuxRE motifs are presented bold and underlined. For GRE- and AuxRE mutated GRAUX-module versions oligos were used in which the ACGT core sequence of the GRE was changed into AAAA and the core sequence of the AuxRE motif (TGTCTC or TGTCCC) in TAAAC or TATCCC, respectively.

## Abbreviations

AC, ACTCAT motif of group S1 bZIP-TFs; ARFs, Auxin Response Factors; AUX/IAAs, AUX/IAA repressor proteins; AuxRE, Auxin Response Element; bZIP, Basic leucine Zipper; GRE, G-box related Element; IAA, Indole-3-acetic acid; MRE, MYB Response Element; TFs, Transcription Factors; TGA, TGA Response Element; wt, Wild type; ZRE, bZIP Response Element.

## Competing interests

The authors declare that they have no competing interests.

## Authors’ contributions

CW and KB wrote the manuscript and interpreted the results. KB wrote the algorithm and KB and DW performed the *cis*-element analysis, JK did the microarray analysis, CW generated reporter constructs and performed transient protoplast transfection experiments. CW, KB and WDL designed the study. WDL and KH revised the manuscript. All authors read and approved the final manuscript.

## Supplementary Material

Additional file 1 **Expression-profiles of auxin-regulated genes from *A. thaliana.*** Number of auxin-regulated genes determined to be regulated and being significantly (α ≤ 0.05), 2-fold up- or down- regulated compared to the control for each time point. Non-redundant genes identified on ATH1 chip ('On Chip') and the number of genes for which a corresponding promoter could be found ('With Prom.') are given. Expression profiles of these genes are shown. Microarray analysis is explained in the Methods section. Click here for file

Additional file 2 **Auxin-regulated genes from *A. thaliana*and*O. sativa.*** List of auxin-regulated genes for each time point ordered in up- and down-regulation categories. Fold induction, Affymetrix identifiers, Locus identifiers and gene descriptions are provided. Click here for file

Additional file 3 **Extended occurrence lists from *A.thaliana*and*O.sativa.*** Extended occurrence lists of enriched motifs and modules from auxin inducible promoters from *Arabidopsis*, rice and selected auxin-related gene families. Enrichment of *cis*-elements in promoters was determined by parameter I, whereas asterisks indicate an enrichment with respect to parameter II. The number of analysed promoters as well as that of promoters containing at least one of the presented motifs or modules is given in parentheses. Presented modules (bipartite, tripartite) exhibit a variable, but maximal spacing of 100 bps between each embedded motif. Sequences and abbreviations of *cis*-elements are given in Table 1. Click here for file

Additional file 4 **Expression-profiles of auxin-regulated genes from O*. sativa.*** Number of auxin-regulated probe sets ('Probes') determined to be regulated and being significantly (α ≤ 0.05), 2-fold up- or down-regulated compared to the control. From those probes, a sub-set could be mapped to a set of non-redundant genes ('OsGI') and for most of them the corresponding promoters could be found ('Promoter'). Expression profiles of these probes are shown. Microarray analysis is explained in the Methods section. Click here for file

Additional file 5 **Auxin-regulated gene family members from *A. thaliana.*** Presented are gene members of auxin-related gene families used in this work, with identifiers and gene descriptions extracted from the TAIR9 functional descriptions and current gene family classifications [36,69,70]. Click here for file
