# Supplementary figures and images for: Bioinformatic cis-element analyses performed in Arabidopsis and rice disclose bZIP- and MYB-related binding sites as potential AuxRE-coupling elements in auxin-mediated transcription
Source: BMC Plant Biol. 2012 Aug 1;12:125. doi: 10.1186/1471-2229-12-125 (PMC3438128; doi:10.1186/1471-2229-12-125)

# Arabidopsis

|            | UP    |       |       |
|------------|-------|-------|-------|
|            | 0.5 h | 1.0 h | 3.0 h |
| With Prom. | 179   | 250   | 398   |
| On Chip    | 180   | 251   | 400   |

  

|            | DOWN  |       |       |
|------------|-------|-------|-------|
|            | 0.5 h | 1.0 h | 3.0 h |
| With Prom. | 26    | 51    | 348   |
| On Chip    | 26    | 53    | 349   |

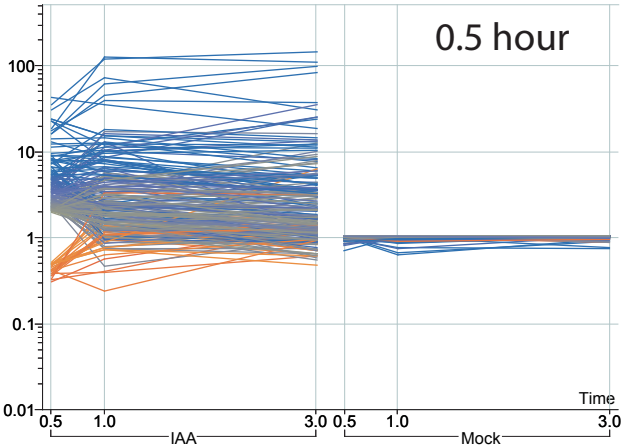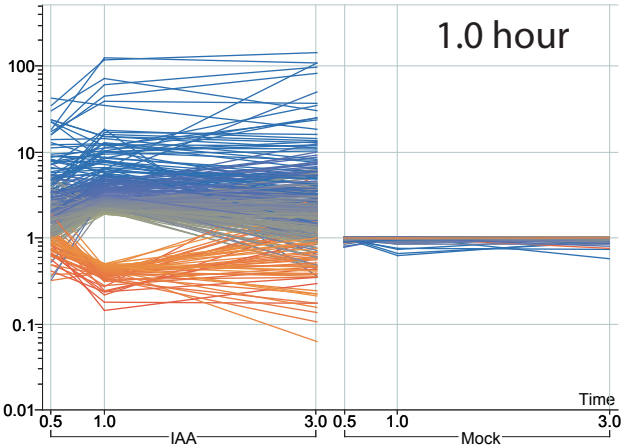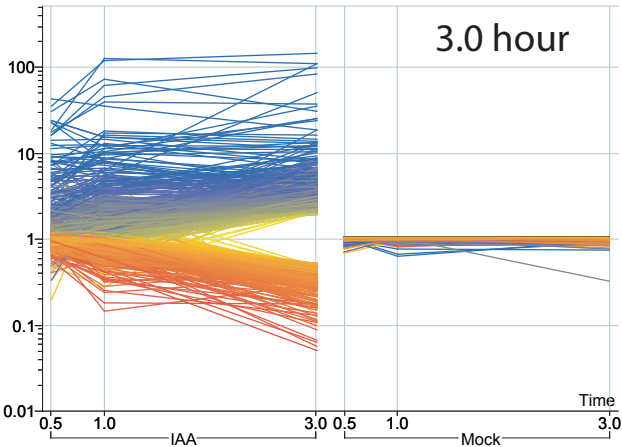

Supplement: Additional file 1 — Expression-profiles of auxin-regulated genes from A. thaliana. Number of auxin-regulated genes determined to be regulated and being significantly (α ≤ 0.05), 2-fold up- or down- regulated compared to the control for each time point. Non-redundant genes identified on ATH1 chip ('On Chip') and the number of genes for which a corresponding promoter could be found ('With Prom.') are given. Expression profiles of these genes are shown. Microarray analysis is explained in the Methods section. [file 1471-2229-12-125-S1.pdf]

# Rice

|          | <u>UP</u> | <u>DOWN</u> |
|----------|-----------|-------------|
| Promoter | 203       | 79          |
| OsGI     | 204       | 80          |
| Probes   | 305       | 126         |

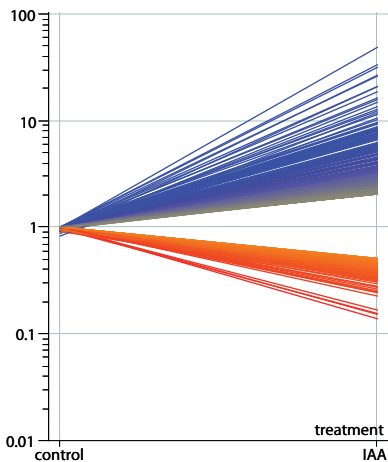

Supplement: Additional file 4 — Expression-profiles of auxin-regulated genes from O. sativa. Number of auxin-regulated probe sets ('Probes') determined to be regulated and being significantly (α ≤ 0.05), 2-fold up- or down-regulated compared to the control. From those probes, a sub-set could be mapped to a set of non-redundant genes ('OsGI') and for most of them the corresponding promoters could be found ('Promoter'). Expression profiles of these probes are shown. Microarray analysis is explained in the Methods section. [file 1471-2229-12-125-S4.pdf]
