# Supplementary material for: Bioinformatic cis-element analyses performed in Arabidopsis and rice disclose bZIP- and MYB-related binding sites as potential AuxRE-coupling elements in auxin-mediated transcription
Source: BMC Plant Biol. 2012 Aug 1;12:125. doi: 10.1186/1471-2229-12-125 (PMC3438128; doi:10.1186/1471-2229-12-125)
Supplement: Additional file 3 — Extended occurrence lists from A.thalianaandO.sativa. Extended occurrence lists of enriched motifs and modules from auxin inducible promoters from Arabidopsis, rice and selected auxin-related gene families. Enrichment of cis-elements in promoters was determined by parameter I, whereas asterisks indicate an enrichment with respect to parameter II. The number of analysed promoters as well as that of promoters containing at least one of the presented motifs or modules is given in parentheses. Presented modules (bipartite, tripartite) exhibit a variable, but maximal spacing of 100 bps between each embedded motif. Sequences and abbreviations of cis-elements are given in Table 1. [file 1471-2229-12-125-S3.docx]

**Extended Table 2: Occurrence list of enriched motifs and modules in early and late auxin-inducible promoters from *A. thaliana***

| **combination** | **ORDER** | **0.5 h UP (179)** |  | **1 h UP (250)** |  | **3 h UP (398)** |
| --- | --- | --- | --- | --- | --- | --- |
| **singles** | 1 | AUX2 (134) | 1 | GRE (172) | 1 | GRE (273) |
|  | 2 | GRE (123) | 2 | MYC2 (136) | 2 | MYC2 (197) |
|  | 3 | AUX1 (93) | 3 | AUX1 (129) | 3 | RY (27) |
|  | 4 | MYC2 (91) | 4 | .. | 4 | .. |
|  | 1* | .. | 1* | AUX2 (181) | 1* | .. |
|  | | | | | | |
| **bipartite** | 1 | GRE-AUX2 (33) | 1 | GRE-MRE2 (122) | 1 | GRE-MRE2 (188) |
|  | 2 | GRE-MRE1 (32) | 2 | GRE-AUX2 (43) | 2 | MRE1-GRE (65) |
|  | 3 | MYC2-AUX2 (27) | 3 | AUX2-GRE (39) | 3 | MYC2-AUX2 (42) |
|  | 4 | AUX2-MYC2 (23) | 4 | MYC2-AUX2 (34) | 4 | GRE-MYC2 (37) |
|  | 5 | GRE-AUX1 (21) | 5 | GRE-AUX1 (32) | 5 | AUX2-MYC2 (35) |
|  | 6 | AUX1-MYC2 (14) | 6 | AUX2-MYC2 (28) | 6 | GRE-AUX1 (31) |
|  | 7 | MYC2-AUX1 (13) | 7 | AUX1-MYC2 (17) | 7 | AUX1-MYC2 (20) |
|  | 8 | MRE2-RY (9) | 8/9 | RY-MRE2 (13) | 8 | RY-MRE2 (18) |
|  | 9 | RY-AC (2) | 8/9 | MYC2-AUX1 (13) | 9 | MRE2-RY (15) |
|  | 10 | .. | 10 | MRE2-RY (11) | 10 | .. |
|  | | | | | | |
| **tripartite** | 1 | GRE-AUX2-MRE2 (22) | 1 | GRE-AUX2-MRE2 (27) | 1 | RY-AC-MYC2 (1) |
|  | 2 | GRE-AUX1-MRE2 (15) | 2 | GRE-AUX1-MRE2 (20) | 2 | MYC2-AC-RY (1) |
|  | 3 | MRE2-GRE-AUX1 (13) | 3 | MRE2-GRE-AUX1 (19) | 3 | .. |
|  | 4 | GRE-AUX2-MRE1 (8) | 4 | GRE-AUX2-MRE1 (9) | 4 | .. |
|  | 5 | GRE-AUX1-MRE1 (6) | 5 | GRE-AUX1-MRE1 (6) | 5 | .. |
|  | 6 | MYC2-AUX2-GRE (5) | 6 | MYC2-TGA-AUX1 (3) | 6 | .. |
|  | 7 | MYC2-AUX1-GRE (4) | 7 | RY-MYC2-AC (1) | 7 | .. |
|  | 8 | MRE1-AUX1-AC (4) | 8 | MRE1-RY-AC (1) | 8 | .. |
|  | 9 | MYC2-TGA-AUX1 (3) | 9 | .. | 9 | .. |
|  | 10 | AC-AUX1-MYC2 (2) | 10 | .. | 10 | .. |
|  | 1* | .. | 1* | GRE-RY-MRE1 (1) | 1* | AUX1-TGA-MRE1 (2) |
|  | 2* | .. | 2* | .. | 2* | .. |

**Extended Table 3: Occurrence list of enriched motifs and modules within auxin-inducible promoters from *O. sativa***

| **combination** | **ORDER** | | **UP (203)** |
| --- | --- | --- | --- |
| **singles** | 1 | | RY (26) |
|  | 1* | | GRE (144) |
|  | 2* | | AUX2 (140) |
|  | 3* | | TGA (130) |
|  | |  | |
| **bipartite** | 1 | | AUX2-GRE (45) |
|  | 2 | | MRE1-GRE (39) |
|  | 3 | | AUX2-MYC2 (26) |
|  | 4 | | AUX1-GRE (21) |
|  | 5 | | RY-MRE2 (14) |
|  | 6 | | AUX1-MYC2 (12) |
|  | 7 | | RY-GRE (7) |
|  | 8 | | RY-MYC2 (5) |
|  | 9 | | RY-AC (3) |
|  | 1* | | TGA-MRE2 (89) |
|  | 2* | | MRE2-TGA (83) |
|  |  | |  |
| **tripartite** | 1 | | AUX2-GRE-MRE2 (25) |
|  | 2 | | MRE2-AUX2-GRE (24) |
|  | 3 | | AUX1-GRE-MRE2 (13) |
|  | 4/5 | | MRE2-AUX1-GRE (12) |
|  | 4/5 | | AUX1-MRE2-TGA (12) |
|  | 6-8 | | GRE-RY-MRE2 (5) |
|  | 6-8 | | AUX1-GRE-MYC2 (5) |
|  | 6-8 | | MRE2-RY-GRE (5) |
|  | 9/10 | | AC-AUX2-MYC2 (4) |
|  | 9/10 | | GRE-MRE2-RY (4) |

**Extended Table 4:** **Occurrence list of enriched *cis*-elements and modules in promoters of auxin-responsive gene families from *A. thaliana***

| **combination** | **ORDER** | **AtGH3 (20)** |  | **AtAUX/IAAs (29)** |  | **AtARFs (23)** |  | **AtSAURS (80)** |
| --- | --- | --- | --- | --- | --- | --- | --- | --- |
| **singles** | 1 | MYC2 (13) | 1 | MYC2 (18) | 1 | .. | 1 | MYC2 (47) |
|  | 2 | .. | 2 | .. | 2 | .. | 2 | .. |
|  | 1* | .. | 1* | AUX2 (21) | 1* | .. | 1* | AUX2 (58) |
|  | 2* | .. | 2* | AUX1 (17) | 2* | .. | 2* | .. |
|  |  |  |  |  |  |  |  |  |
| **bipartite** | 1 | MYC2-AUX2 (5) | 1 | MYC2-GRE (7) | 1 | .. | 1 | MYC2-AUX2 (17) |
|  | 2 | MYC2-AUX1 (3) | 2 | AUX2-MYC2 (6) | 2 | .. | 2 | AUX2-MYC2 (14) |
|  | 3 | .. | 3/4 | AUX1-MYC2 (5) | 3 | .. | 3 | AUX1-MYC2 (9) |
|  | 4 | .. | 3/4 | MYC2-AUX2 (5) | 4 | .. | 4 | MRE1-RY (2) |
|  | 5 | .. | 5-7 | RY-MYC2 (1) | 5 | .. | 5 | .. |
|  | 6 | .. | 5-7 | RY-AC (1) | 6 | .. | 6 | .. |
|  | 7 | .. | 5-7 | MYC2-RY (1) | 7 | .. | 7 | .. |
|  | 1* | AUX2-AC (1) | 1* | GRE-RY (1) | 1* | .. | 1* | .. |
|  |  |  |  |  |  |  |  |  |
| **tripartite** | 1/2 | GRE-AUX2-MRE1 (3) | 1 | MRE2-AUX2-GRE (6) | 1 | MYC2-AUX1-AC (1) | 1 | AC-MRE2-RY (2) |
|  | 1/2 | AUX2-MRE1-GRE (3) | 2 | MRE1-AUX2-GRE (3) | 2 | .. | 2/3 | MRE1-RY-AC (1) |
|  | 3-10 | AUX2-TGA-MRE1 (2) | 3-6 | MRE1-GRE-AUX1 (2) | 3 | .. | 2/3 | RY-AC-MRE2 (1) |
|  | 3-10 | GRE-MRE1-AUX2 (2) | 3-6 | MRE1-AUX1-GRE (2) | 4 | .. | 4 | .. |
|  | 3-10 | GRE-MYC2-AUX1 (2) | 3-6 | AUX1-MYC2-GRE (2) | 5 | .. | 5 | .. |
|  | 3-10 | GRE-MYC2-AUX2 (2) | 3-6 | AUX2-MYC2-GRE (2) | 6 | .. | 6 | .. |
|  | 3-10 | MYC2-AUX2-TGA (2) | 7-10 | GRE-RY-MRE2 (1) | 7 | .. | 7 | .. |
|  | 3-10 | MYC2-AUX2-GRE (2) | 7-10 | GRE-MRE2-RY (1) | 8 | .. | 8 | .. |
|  | 3-10 | GRE-AUX1-MRE1 (2) | 7-10 | MYC2-RY-AC (1) | 9 | .. | 9 | .. |
|  | 3-10 | AUX1-MRE1-GRE (2) | 7-10 | RY-AC-MYC2 (1) | 10 | .. | 10 | .. |
|  | 1* | .. | 1* | .. | 1* | .. | 1/2* | MRE2-AUX2-TGA (1) |
|  | 2* | .. | 2* | .. | 2* | .. | 1/2* | AUX2-MRE2-TGA (1) |
